# Structural dynamics and determinants of 2-aminoadenine specificity in DNA polymerase DpoZ of vibriophage ϕVC8

**DOI:** 10.1093/nar/gkab955

**Published:** 2021-11-09

**Authors:** Dariusz Czernecki, Haidai Hu, Filippo Romoli, Marc Delarue

**Affiliations:** Unit of Architecture and Dynamics of Biological Macromolecules, CNRS UMR 3528, 25-28 rue du Docteur Roux, Institut Pasteur, 75015 Paris, France; Sorbonne Université, Collège Doctoral, ED 515, 75005 Paris, France; Unit of Architecture and Dynamics of Biological Macromolecules, CNRS UMR 3528, 25-28 rue du Docteur Roux, Institut Pasteur, 75015 Paris, France; Unit of Architecture and Dynamics of Biological Macromolecules, CNRS UMR 3528, 25-28 rue du Docteur Roux, Institut Pasteur, 75015 Paris, France; Unit of Architecture and Dynamics of Biological Macromolecules, CNRS UMR 3528, 25-28 rue du Docteur Roux, Institut Pasteur, 75015 Paris, France

## Abstract

All genetic information in cellular life is stored in DNA copolymers composed of four basic building blocks (ATGC-DNA). In contrast, a group of bacteriophages belonging to families *Siphoviridae* and *Podoviridae* has abandoned the usage of one of them, adenine (A), replacing it with 2-aminoadenine (Z). The resulting ZTGC-DNA is more stable than its ATGC-DNA counterpart, owing to the additional hydrogen bond present in the 2-aminoadenine:thymine (Z:T) base pair, while the additional amino group also confers resistance to the host endonucleases. Recently, two classes of replicative proteins found in ZTGC-DNA-containing phages were characterized and one of them, DpoZ from DNA polymerase A (PolA) family, was shown to possess significant Z-vs-A specificity. Here, we present the crystallographic structure of the apo form of DpoZ of vibriophage ϕVC8, composed of the 3′-5′ exonuclease and polymerase domains. We captured the enzyme in two conformations that involve the tip of the thumb subdomain and the exonuclease domain. We highlight insertions and mutations characteristic of ϕVC8 DpoZ and its close homologues. Through mutagenesis and functional assays we suggest that the preference of ϕVC8 DpoZ towards Z relies on a polymerase backtracking process, more efficient when the nascent base pair is A:T than when it is Z:T.

## INTRODUCTION

All known biological entities on Earth encode their genetic information in nucleic acids. Whereas cellular life uses for that purpose DNA, some viruses extend the information storage capabilities to its chemical analogue, RNA. Although the genomic material can be decorated in a variety of ways, conferring additional, epigenetic information or providing resistance against nucleases (1–3), this cannot interfere with the ability of the nucleic acid to duplicate. Replication is ensured by the complementarity of the four building blocks (nucleobases): adenine (A), thymine (T), guanine (G) and cytosine (C). Base pairs A:T and G:C are maintained by hydrogen bonding on the so-called Watson-Crick edge of the bases. Due to its crucial character, chemical modifications of the bases on this edge are strongly disfavoured.

The only known exception to the universality of A:T and G:C base-pairing is a group of bacteriophages from families *Siphoviridae* and *Podoviridae*, such as cyanophage S-2L, vibriophage ϕVC8 and acinetophage SH-Ab 15497. Their original genetic material is completely devoid of adenine (4–6). Instead, their DNA contains 2-aminoadenine (2,6-diaminopurine or Z) which specifically pairs with genomic thymine, and encodes enzymes necessary both for the biosynthesis of dZTP and the removal of dATP (6–9). The additional chemical group on the Watson-Crick edge preserves the purine–pyrimidine pairing and confers an additional (third) hydrogen bond to the Z:T pair compared to the A:T pair. This results in a higher melting point of S-2L ZTGC-DNA compared to ATGC-DNA (10) as well as in additional endonuclease resistance (11). Interestingly, due to mutual compatibility of A and Z with T, ZTGC-DNA is readily converted into ATGC-DNA under standard amplification reactions; the detection of ZTGC-DNA remains nevertheless possible with single-molecule sequencing methods (6).

While nucleic acids could have originally been endowed with the ability to auto-catalyse their replication (12), all modern organisms and viruses have evolved specialized proteins called RNA and DNA polymerases (Pol) to efficiently duplicate their genomes. DNA polymerases are classified into different families: A, B, C, D, X, Y, RT and PrimPol (13–15). ZTGC-DNA phages usually encode PolA and PrimPol, families that were shown to be implicated in plasmidic or viral DNA replication (16,17). Although the sole polymerase of S-2L (a PrimPol) is unspecific towards Z (7), other phages do rely on highly Z-specific PolA enzymes called DpoZ (5).

Phylogenetically speaking, DpoZ are split into two distinct subfamilies: ϕVC8-like and Wayne-like (5). Both harbour 5′-3′ polymerase and 3′-5′ exonuclease domains and display a strong exonuclease activity. ϕVC8-like DpoZ were studied in detail for phages ϕVC8 and SH-Ab 15497, showing catalytic efficiencies greater by 1–2 orders of magnitude with dZTP than with dATP (5). This would make them useful tools for controlling the replication of synthetic ZTGC-DNA organisms that could be generated with a set of 3 genes implicated in 2-aminoadenine metabolism (9).

Importantly, phages S-2L (with PrimPol) and ϕVC8-like (with DpoZ) harbour in their genomes a well-conserved gene for a nucleotide phosphohydrolase (DatZ) (5–7). In S-2L, we showed that DatZ removes the triphosphate tail of dATP (and of no other dNTP), rendering it unusable as a substrate for a PrimPol DNA polymerase. DatZ of ϕVC8-like phage SH-Ab 15497 was shown to function as a dATPase as well (6). Whereas this enzyme is the main factor of adenine exclusion in S-2L (7), in phages of the ϕVC8 clade it may act in complement with DpoZ. In the Wayne-like clade (5) a gene homologous to deoxyuridine phosphatases may fulfil a role analogous to DatZ, and/or to another phosphatase (MazZ) involved in Z synthesis (6,9).

Here, we solve the crystallographic structure of ϕVC8 DpoZ in two conformational states, called thumb-exo open and closed forms, that reveal previously undescribed movements of a novel extension in the exonuclease domain and of the polymerase thumb subdomain. We further identify 3 new insertions in the palm and fingers subdomains, and map onto the model DpoZ-specific residues that depart from other PolA in highly conserved regions (motifs). We assess the role of 3′-5′ exonuclease domain in 2-aminoadenine specificity, combining functional results of wild-type ϕVC8 DpoZ and mutants of crucial residues in this domain with the structural data. Finally, we offer an explanation for a plausible mechanism behind the Z-vs-A preference, coherent with all available results. Altogether, this structural framework will be useful to design mutants with even better specificity, for instance by directed evolution.

## MATERIALS AND METHODS

### Protein expression and purification

For structural studies, the wild-type gene of ϕVC8 DpoZ described in (18) was cloned into a modified pET-47b expression vector as described in (5), which was provided by F. Jaziri and V. Pezo. For functional assays, a synthetic version of ϕVC8 DpoZ was optimized for *Escherichia coli*, synthesized using ThermoFisher's GeneArt service and cloned into modified pRSF1-Duet vector with a 14-histidine tag (19) (Supplementary Table S1). Cloning was performed using New England Biolabs and Anza (Thermo Fisher Scientific) enzymes. Mutagenesis of DpoZ was done using designed oligonucleotides and QuikChange II Site-Directed Mutagenesis Kit (Agilent). *E. coli* BL21-CodonPlus (DE3)-RIPL cells (Agilent) were separately transformed with the engineered plasmids. Bacteria were cultivated at 37°C in LB medium with appropriate antibiotic selection (kanamycin and chloramphenicol), and induced at OD = 0.6–1.0 with 0.5 mM IPTG. After incubation overnight at 20°C, cells were harvested and homogenized in suspension buffer: 20 mM Tris–HCl pH 6.8, 400 mM NaCl, 5 mM imidazole. After sonication and centrifugation of bacterial debris, corresponding lysate supernatants were supplemented with Benzonase (Sigma-Aldrich) and protease inhibitors (Thermo Fisher Scientific), 1 μl and 1 tablet per 50 ml, respectively. DpoZ protein was isolated by purification of the lysate on Ni-NTA column (suspension buffer as washing buffer, 500 mM imidazole in elution buffer). Fractions of interest were further diluted to 200 mM NaCl and repurified on HiTrap Heparin column (1 M NaCl and no imidazole in the elution buffer). Proteins were further purified on Superdex 200 10/300 column with 20 mM Tris–HCl pH 6.8, 400 mM NaCl—as we observed that high salt concentration increased DpoZ stability. All purification columns were from Life Sciences. Protein purity was assessed on an SDS gel (BioRad). The enzymes were concentrated to 10–15 mg ml^–1^ with Amicon Ultra 30k MWCO centrifugal filters (Merck), flash frozen in liquid nitrogen and stored directly at −80°C, with no glycerol added.

### DNA polymerase assays

Fluorescence polymerase activity tests were executed in 20 mM Tris–HCl pH 7 and 50 mM MgCl_2_, with 3 μM of dT_24_ overhang DNA template, 1 μM of FAM 5′-labeled DNA primer (Supplementary Table S2), varying concentration of dATP or dZTP and 0.83 μM (0.06 mg ml^–1^) of DpoZ constructs (5 min of incubation at 37°C in 100 μl volume). The Klenow polymerase used as a control was at 5 U in 50 μl (10 min incubation) and was purchased from Takara Bio.

Before adding the protein, DNA was hybridized by heating up to 95°C and gradually cooling to reaction temperature. Reactions were terminated by adding two volumes of a buffer containing 10 mM EDTA, 98% formamide, 0.1% xylene cyanol and 0.1% bromophenol blue, and stored at 4°C. Products were preheated at 95°C for 10 min, before being separated with polyacrylamide gel electrophoresis and visualised by FAM fluorescence or radioactivity on Typhoon FLA 9000 imager. All oligonucleotides were ordered from Eurogentec, chemicals from Sigma-Aldrich, dATP from Fermentas (Thermo Fisher Scientific) and dZTP from TriLink BioTechnologies.

Intensities of the nucleic acid bands corresponding to fully extended primers were quantified with ImageJ (20); mid-points of dNTP concentrations needed for product saturation were determined by fitting a sigmoidal function to the data.

### Crystallography and structural analysis

Crystallization conditions were screened using the sitting drop technique on an automated crystallography platform (21) and were reproduced manually using the hanging drop method with ratios of protein to well solution ranging from 1:2 to 2:1. DpoZ was screened at 10–15 mg ml^–1^ at 18°C. Several small crystals grew during several days in 1 mM cobalt hexamine and 25% (v/v) isopropanol (100%) buffered with 100 mM HEPES pH 7. They diffracted to a resolution limit of about 8 Å. The crystals were then optimized by three rounds of micro-seeding in a solution containing 12.5 mg ml^–1^ of the protein, 300 mM ammonium citrate and 12% w/v PEG 3350, selected by further manual screening. Final DpoZ crystals were soaked for several seconds with 30% (v/v) glycerol and 70% crystallization buffer, before being frozen in liquid nitrogen. Crystallographic data was collected at the Soleil synchrotron in France (beamlines PX1 and PX2) and processed by XDS (22) with the XDSME (23) pipeline. The structure was solved by molecular replacement with *Thermus aquaticus* Pol I (Klentaq) model (PDB ID: 1KTQ, 25% identity to DpoZ) and refined in Phenix (24) using Coot for manual reconstruction (25) (Supplementary Table S3).

### Sequence and structure alignments and analysis

Structures homologous to DpoZ available in PDB were identified using Dali server (26). Dali was further used for pairwise RMSD determination and geometry analysis. Structural multialignment of DpoZ and PolA enzymes was calculated with PROMALS3D (27): due to an important structural divergence across these polymerases, its accuracy is not absolute in regions varying in length and/or conformation, but it was verified to correctly cover the regions of interest. Graphical display of multiple sequence alignments was prepared with ESPript 3 (28). Protein structures were analysed with Chimera (29) or Pymol (30). Sequences of close relatives of *dpoZ* were identified by BLAST searches (31). Normal mode analysis was calculated using the online interface NOMAD-Ref using default parameters (32).

## RESULTS

### Expression and purification of functional ϕVC8 DpoZ

The synthetic gene of ϕVC8 DpoZ was cloned in *E. coli* cells with an additional N-terminal 6- or 14-histidine tag. We overexpressed the gene and performed a three-step purification of the product. Final fractions visualised on an SDS-PAGE gel showed a single band corresponding to the protein of expected molecular weight (Supplementary Figure S1). Dynamic light scattering (DLS) quality assay showed no protein aggregates in solution.

**Figure 1.**
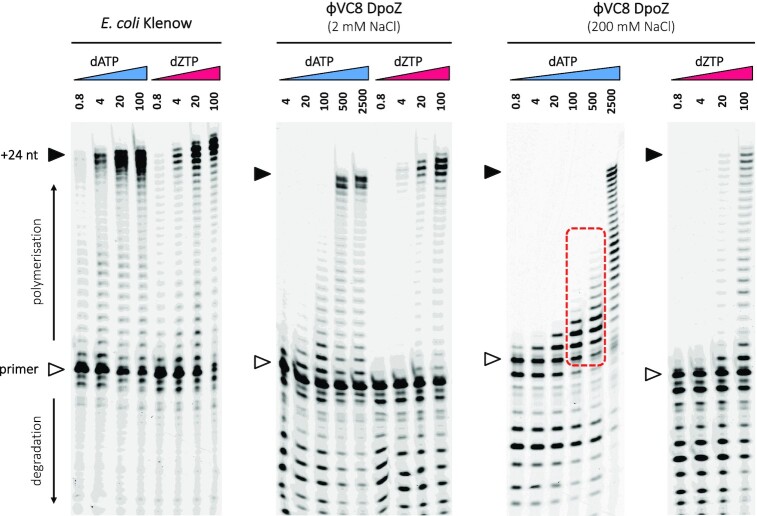
Polymerase activity for E. coli Pol I (Klenow fragment) and ϕVC8 DpoZ, using the primer extension assay visualized on polyacrylamide gels. The templating substrate is dT_24_. Bands above the primer line (white arrow) represent primer elongation—this includes the full-length product (black arrow). In contrast, bands below the primer line show degradation through intrinsic exonuclease activity of PolA enzymes. For DpoZ, assays were made at two different (high or low) concentrations of NaCl, as specified above the gels. The concentration of nucleotides—dATP (blue) or dZTP (magenta)—is indicated above each lane, in μM. Dotted red rectangle indicates adenine incorporation events detected in physiological conditions.

To confirm the activity and specificity of the purified ϕVC8 DpoZ towards 2-aminoadenine, we incubated the enzyme with a primed polythymine (dT_24_) template and with various concentrations of dATP or dZTP. Pure DpoZ indeed displays Z-over-A specificity (Figure 1). Complete strand synthesis with dZTP as a substrate occurs in concentrations between 1 and 2 orders of magnitude lower than for an analogous reaction with dATP. Although the overall polymerase efficiency of DpoZ decreases at higher NaCl concentrations, its selectivity is not affected. Importantly, we could determine that with substrate dATP and salt concentrations matching physiological conditions in bacteria (175–181 μM of intracellular dATP (33), 100–300 mM of NaCl (34)), DpoZ regularly incorporates 1–6 successive adenine bases (4–25% of the template's length) over the course of the reaction, as indicated on the figure.

We conclude that although DpoZ undeniably shows strong exonuclease activity, it does not fully prevent polymerisation with dATP as a substrate – if dATP is available, the enzyme will most probably incorporate trace amounts of adenine into nascent DNA. However, the dATPase activity of DatZ, encoded in the ϕVC8 genome as well (6,7), can be invoked to supplement the role of DpoZ and therefore explain the total absence of genomic adenine in this and related phages.

### Structure of DpoZ caught in two states: thumb-exo open and closed

Having confirmed the activity of the purified DpoZ, we started crystallization assays. The protein was not prone to crystallize, possibly due to the known high flexibility of the enzyme, common among family A DNA polymerases (35). Through extensive screening and optimization, we could eventually obtain single crystals that diffracted to 2.8 Å.

**Figure 2.**
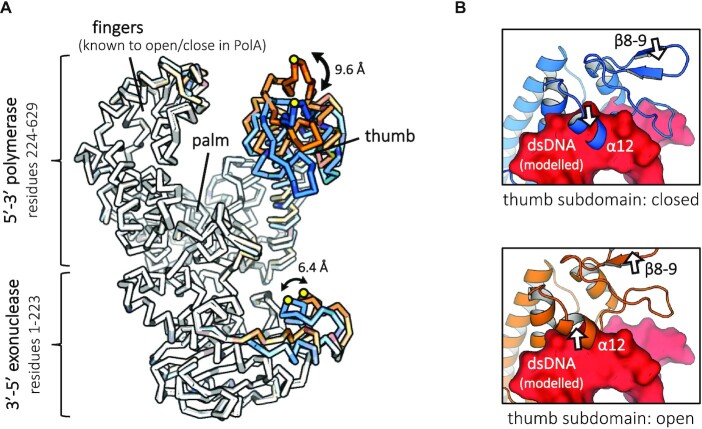
Crystal structure of ϕVC8 DpoZ capturing movements previously undescribed in PolA. (**A**) The two molecules constituting the crystallographic asymmetric unit in ribbon representation, superimposed. Chain A in a ‘thumb-exo closed’ conformation is coloured in light blue, chain B in a ‘thumb-exo open’ state—in orange. Colour saturation scales with RMSD between corresponding C_α_ atoms in the two states, highlighting the simultaneous movement of the thumb subdomain and part of the exonuclease domain. The amplitudes of the movement are quantified at places with the greatest positional shift (residues K162 and G276). (**B**) Movement of the thumb subdomain (white arrows) impacts the accessibility for the primer:template dsDNA (surface view in red, modelled after PDB ID: 1LV5). In the closed form (above), α-helix α12 (residues 299–303) clashes with the nucleic acid. In the open form (below), the clash is vastly reduced, allowing for the proper positioning of the DNA substrate with only minimal further accommodation of the helix. The movement of other thumb elements, such as the large hinge motion of beta turn β8–9, does not interfere with DNA binding.

The structure of DpoZ, with two copies in the asymmetric unit, was solved with molecular replacement, using the Klenow fragment of *T. aquaticus* Pol I (PDB ID: 1KTQ) as a starting model (Supplementary Table S3). The enzyme exhibits the typical fold of the PolA family (Figure 2A), with easily identifiable polymerase (pol) and 3′-5′ exonuclease (exo) domains (36). The former resembles a right hand ready to grip DNA, with its palm subdomain carrying the catalytic site and both thumb and finger subdomains clamping and directing the DNA substrate. Overall, DpoZ superimposes very well with *E. coli* (PDB ID: 1D8Y) and *T. aquaticus* (PDB ID: 1KTQ) Pol I Klenow fragments (RMSD of 3.0–3.5 Å over 496–540 C_α_ atoms).

The two molecules contained in the asymmetric unit of the DpoZ crystal represent different conformations. The transition between the two forms of the molecule involves mainly two subdomains moving mostly as rigid-bodies (Figure 2A; Supplementary Movie S1): the thumb subdomain known to hold the nascent double-stranded DNA (36) and an extension in the exonuclease domain, present in a reduced form in only a few other PolA structures (see below). This is different from the typical open and closed forms observed in other PolA structures, which involve mainly the fingers subdomain and notably its helix O in the polymerase module, but no change in the thumb or exonuclease parts (37,38). Thus, the two DpoZ conformations are hereafter referred to as the ‘thumb-exo’ open and ‘thumb-exo’ closed states. Their RMSD is 2.0 Å for the whole chain, 1.8 Å for the polymerase domain and 1.4 Å for the exonuclease module. 99% of the molecule was constructed for the closed conformation, and 96% for the open one, leaving only a few residues unresolved in the final electron density map.

In the DpoZ crystal, the mobile part of the thumb subdomain in both states contacts the rigid side of another DpoZ molecule (on the fingers/fingers-exo interface side), whereas the mobile part of the exonuclease domain is entirely solvent-exposed. Although crystallographic packing could have influenced the thumb's displacement, the structure still shows here its capacity for movement. Importantly, modelling of the dsDNA substrate in the polymerase domain of DpoZ indicates that the thumb subdomain in its closed state prevents the correct binding of the nucleic acid substrate (Figure 2B).

The conformational transition between the thumb-exo open and closed states is well explained by a handful of low-frequency normal modes (Supplementary Figure S2) that affect large parts of the protein (39). The fact that the elastic network-based coarse-grained model (40) successfully predicts DpoZ structural transition means that the motions in the thumb subdomain and in the exonuclease domain depend inherently on the shape of the structure (structure-encoded movements), and not the atomic details conferred by specific residues. The model therefore predicts that such motions should be possible in other PolA polymerases, provided they share similar structural features in the mobile regions.

### Insertions characteristic of ϕVC8-like DpoZ

**Figure 3.**
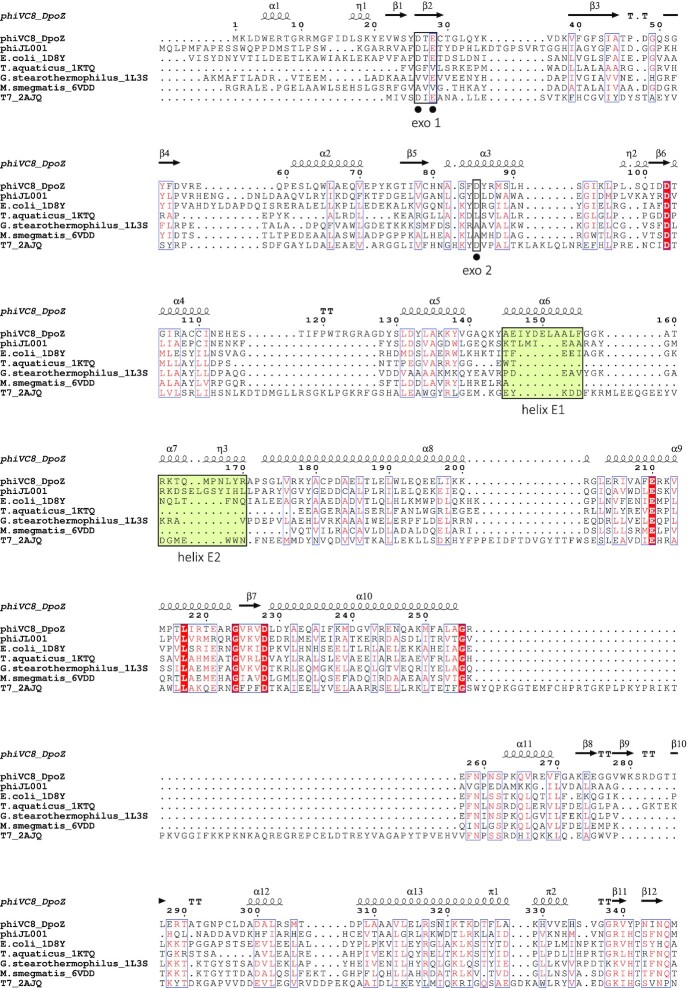

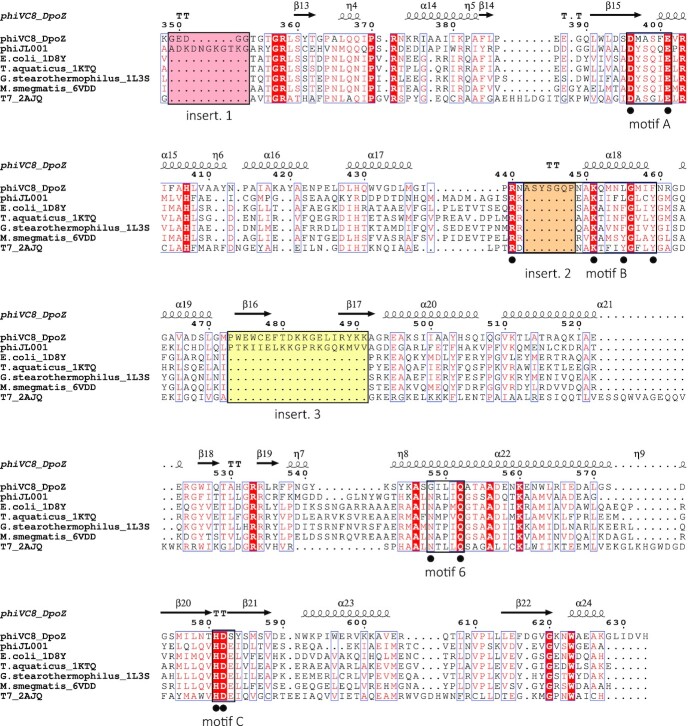
Structural multialignment between ϕVC8 DpoZ and large (Klenow) fragments of other structurally known prokaryotic and viral representatives of PolA family (64–68). This structural alignment was complemented with a close homologue of DpoZ, PolA of phage ϕJL001 which is missing the genes for 2-aminoadenine synthesis (42). Secondary structure and numbering above the alignment refers to ϕVC8 DpoZ. Organism names are shown on the left. Black boxes represent conserved motifs in the exonuclease and polymerase domains, respectively; black dots underneath point to residues with a known function in the catalytic activity. Helix α18 in motif B is typically referred to as helix O in other PolA, as named originally in Klenow Pol I (see Figure 4B). Residues in white with a red background are strictly conserved residues. Lime boxes indicate the exonuclease extensions, while red, orange and yellow boxes delimit insertions specific to the ϕVC8-like DpoZ clade. For clarity reasons, eukaryotic PolA structures were omitted (Pol gamma, Pol nu, Pol theta, apicoplast PolA); Pol gamma and Pol nu already display an insertion around residues 278–285.

Comparing to the structures of other PolA family members (PDB IDs: 1D8Y, 1KTQ, 1L3S, 2AJQ, 3IKM, 4X0Q, 4XVK, 5DKU, 6VDD), the model of ϕVC8 DpoZ presents three unique insertions. They are made apparent in a structural multialignment, that for better readability we present here for ϕVC8 DpoZ and other viral and prokaryotic PolA only (i.e. without the eukaryotic ones) (Figure 3); we note, however, that these insertions are absent in eukaryotic PolA as well. The structural multialignment was supplemented with the sequence of phage ϕJL001 DNA polymerase, which has been already described as related to ϕVC8-like DpoZ (from vibriophage VP2) (41): although the genome of ϕJL001 lacks genes involved in Z biosynthesis (6,8,9,42), its polymerase possesses two of the insertions characteristic of ϕVC8-like DpoZ, and one of their characteristic point mutation (Figure 3, see below).

**Figure 4.**
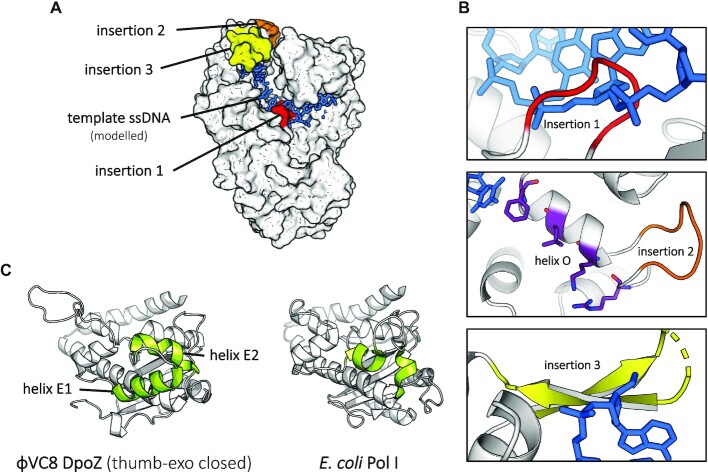
Insertions of ϕVC8-like DpoZ compared to other PolA structures. (**A**) The three insertions (red, orange and yellow, as in Figure 3) conserved in ϕVC8-like DpoZ subfamily, are mapped onto the enzyme in the closed form (white) in surface representation. Additionally, a template ssDNA strand (blue) has been modelled from the ternary complex of *G. stearothermophilus* Pol I (PDB ID: 1LV5 (44)). (**B**) A close-up on insertions 1, 2 and 3 in ribbon-stick representation. The first insertion (red) clashes with the template in the dsDNA region, suggesting its rearrangement in presence of DNA substrate. The second insertion (orange) interrupts the helix O (helix α18 in Figure 3), but does not displace the functional residues constituting motif B (purple). The third insertion (yellow) is in close contact with the template ssDNA strand. (**C**) The mobile element of the 3′-5′ exonuclease domain in ϕVC8 DpoZ (left, helices in lime green) and its equivalent in *E. coli* Pol I (right; PDB ID: 1D8Y (46)). Two helical fragments between helices E and F, normally quite short and referred to as helix E1 and helix E2 here, are visibly extended in DpoZ.

The three insertions conserved in the ϕVC8-like DpoZ subfamily take the form of external loops of several to about twenty residues, scattered around the polymerase domain's active site (Figure 4A). Following the canonical helix-strand numbering of *E. coli* Pol I (43), they are located between strands 7 and 8 in the palm subdomain (DpoZ strands β12 and β13, residues G349–G353); on the helix O in the fingers subdomain (DpoZ helix α18, residues A442–P448); and between helices O1 and P on the very tip of the fingers subdomain (helices α19 and α20, residues P473–K491)—see Figure 3. The third and longest insertion is only partially visible in the electron density map of ϕVC8 DpoZ, indicating its highly flexible nature in the absence of the DNA substrate.

Due to their location, insertions 1 and 3 probably interact with dsDNA substrate, modelled here from another PolA structure (44): they would contact the template strand in the primer–template DNA duplex and in the ssDNA regions, respectively (Figure 4B). Interestingly, the purely sequence-based multialignment of ϕVC8 DpoZ with *E. coli* Pol I cannot predict the presence of the second insertion (5), which adds a loop inside the movable helix O in the fingers subdomain. As this helix dynamically interacts with the dNTP substrate (45) and reacts to deformations caused by mismatched nucleotides (38), insertion 2 may influence DpoZ sensitivity towards nascent base pairs by impacting the mobility of the helix.

As for the exonuclease domain, the only structural element participating in the thumb-exo open to closed conformational transition is located between the canonical helices E and F (Figure 4C). Although this part is quite divergent or even absent in other PolA structures such as in *T. aquaticus* Pol I (Figure 3), it is present in some of them, including Pol I of *E. coli* and phage T7. Nevertheless, in DpoZ this region is comparatively more expanded and folded predominantly in alpha helices—their length is increased 2.5 times comparing to *E. coli* Pol I (PDB ID: 1D8Y (46)). For naming consistency, we will refer to these mobile non-canonical helices as helices E1 (residues A145–F155) and E2 (residues R161–R170).

Finally, the exonuclease domain hosts an insertion between helices α4 and α5, which is however also present in T7 DNA polymerase. It is interesting to note that a recent article mentioned this region as probably implicated in shuttling the DNA primer's 3′-end between the pol and exo sites (47) (see Discussion and PDB 2AJQ).

### Sequence motifs of the polymerase and exonuclease catalytic sites

Similarly to all other PolA polymerases, crucial sequence motifs of ϕVC8 DpoZ follow the general pattern of this family (48–50) (Figure 3). This includes the universally conserved HD catalytic dyad in Motif C, crucial for polymerase activity (36,51). We note that some other sequences shown in the multialignment missing crucial catalytic residues in the exo domain (in motifs called ‘exo 1’ and ‘exo 2’) are known to represent proteins lacking the proofreading activity (52–54). Our structural alignment reveals that the arginine residue necessary for stabilization of the incoming dNTP’s γ-phosphate (55) and non-mutable in *T. aquaticus* Pol I (56) is in fact structurally conserved, as the insertion 2 disrupting the motif B on helix O leaves arginine's position R440 intact (Figure 4B).

Departures from the conservation profile for classical polymerase motifs include residues L455 and F459 in motif B, residue G548 in motif 6 and residue S583 in motif C - with the exception of the latter, all these mutations are characteristic of the ϕVC8-like DpoZ clade. These residues are located in the active site of the polymerase domain or its immediate vicinity—the second sphere of interactions (Supplementary Figure S3). However, residues corresponding to L455, G548 and S583 are known to exist in other PolA from phages missing the Z-cluster necessary for 2-aminoadenine synthesis: these residues are found, respectively and among others, in the polymerases of phage ϕJL001 (42), vibriophage vB_VspP_pVa5 (57) and phage T5 (58). Furthermore, residues L455 and G548 do not appear in any member of another subfamily of DpoZ, Wayne-like, which are selective towards dZTP as well (5). The only characteristic mutation shared by both clades of DpoZ is F459 in helix O, a mutation from the otherwise strictly conserved tyrosine acting as a steric gate distinguishing between dNTPs and NTPs (59). However, a conservative Tyr-to-Phe mutant of *T.aquaticus* Pol I was nevertheless shown to retain the activity *in vivo* in bacteria devoid of the Z-cluster, complementing the inactive native enzyme (56).

To summarize, the specificity of ϕVC8-like DpoZ is unlikely to be explained by a single mutation in the polymerase domain, as all the atypical residues absent in classical polA motifs but present in ϕVC8 DpoZ appear in at least one unrelated polymerase functional with ATGC-DNA. Instead, such specificity may arise from a complicated interplay of multiple residues and insertions around the catalytic site. In such case, the protein would rather be sensitive to the overall stability properties of the Z:T-vs-A:T base pairs, rather than directly to the presence or absence of the 2-amino group.

### Involvement of helices E1 and E2 in the exonuclease activity

**Figure 5.**
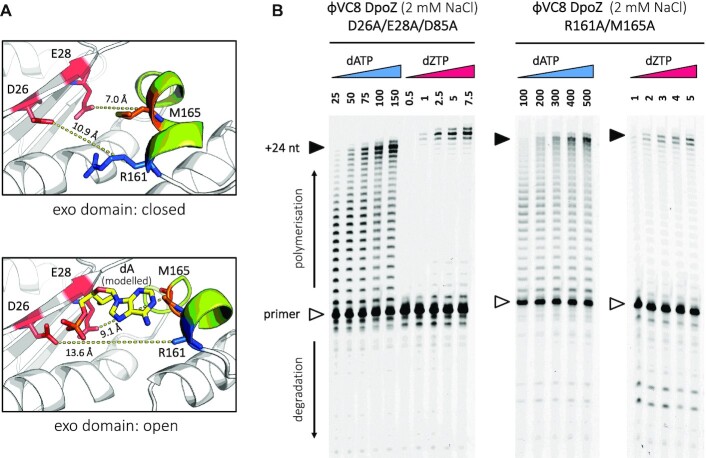
Rearrangement of the ϕVC8 DpoZ exonuclease catalytic pocket and polymerase activity assays for two exonuclease mutants. (**A**) A close-up on the residues of interest showed in stick representation around the exo catalytic site, in the thumb-exo closed (top) and open (bottom) conformations. The distances between catalytic residues D26 and E28 and C_β_ atoms of residues R161 (blue) and M165 (orange) protruding from helix E2 are shown in yellow dashed lines. In the open state, residues R161 and M165 appear to be in contact with a dA nucleotide substrate (yellow) modelled in the exo catalytic site (using PDB ID: 2KFN), even if their side-chain cannot be determined with certainty in the electron density map (see Supplementary Figure S3). In contrast, dA cannot be accommodated in the catalytic pocket in the closed state. (**B**) The triple exonuclease mutant (D26A/E28A/D85A) and helix E2 mutant (R161A/M165A) were subjected to polymerase assays in presence of 2 mM NaCl. The image is annotated as in Figure 1. Both mutants show much lower exonuclease activity compared to the wild type.

Because helices E1 and E2 constitute an unusual mobile piece of the 3′-5′ exonuclease domain, we hypothesized that they could contribute to the proofreading activity of DpoZ. Indeed, modelling a leaving nucleotide in the exo catalytic site in the thumb-exo open conformation showed that residues R161 and M165 of the helix E2 could contact the nucleobase (Figure 5A). Interestingly, although their side-chains are unstructured in the absence of this leaving nucleotide (Supplementary Figure S3), in the closed state these residues are much closer to the core of the exonuclease domain, assuming a particular conformation which blocks the active site from accepting a nucleotide. Also, they are overall well conserved in other DpoZ from ϕVC8-like subfamily: R161 can vary to lysine, while M165 varies to isoleucine and alanine. These conservative mutations preserve the chemical character of the exposed residues, allowing the possibility of their involvement in a common exonuclease mechanism.

To assess if the above residues are indeed relevant for the exonuclease activity, we tested their combined mutation (R161A/M165A) in our polymerase assays (Figure 5B). As a control, we also assessed the activity of a triple DpoZ mutant (D26A/E28A/D85A), where the mutation of three catalytic residues should completely eliminate any trace of the proofreading activity of the enzyme.

Indeed, DpoZ D26A/E28A/D85A showed no significant exonuclease activity compared to the wild-type enzyme (Figure 1). Similarly to the single mutant (D85A) (5), the triple mutant retains dZTP specificity during DNA biosynthesis, forming the full-length product at much lower concentrations of dZTP than dATP. However, compared to the wild-type DpoZ, it also requires a reduced concentration of both dNTPs for the complete product synthesis. This is reflected in the titration of the full-length product by the dNTP substrate and was analysed quantitatively: for dATP, the mid-point of full-length product saturation decreases from 449 μM (±36) for the wild-type to 75 μM (±9) for the exonuclease-deficient mutant. Likewise, for dZTP, it is reduced *by the same factor*, from 15.6 μM (±1.6) to 2.1 μM (±0.3).

DpoZ R161A/M165A performed similarly to the triple exonuclease mutant, showing no significant degradation of the primer. Here, the mid-points of full-length product saturation were measured to be 342 μM (±31) for dATP, and 1.9 μM (±0.5) for dZTP.

In summary, the A-vs-Z ratio of nucleotide concentrations needed for a complete primer extension stays unchanged in the wild-type DpoZ and its triple exonuclease mutant that suppresses the proofreading activity. As for the mobile region containing helices E1 and E2, the R161A/M165A mutation has a detrimental effect on the exonuclease activity. It displays intermediate polymerization efficiencies with dATP and dZTP when compared to DpoZ wild-type and the exonuclease mutant, with the paradoxical and unexpected net effect of a higher Z-vs-A selectivity compared to the wild-type.

## DISCUSSION

From a structural point of view, explaining the specificity of Z-vs-A of DpoZ requires an in-depth analysis to identify features conserved in close homologues but absent in all other PolA polymerases. Indeed, our crystal data for ϕVC8 DpoZ provides a structural context to pinpoint where this specificity could reside in the enzyme, allowing to ask how its distinctive features relate to other polymerases without any Z-vs-A specificity. In the case of the extended exonuclease element and the insertion 2, their precise structural description clearly goes beyond the information contained in the multiple sequence alignment. Strikingly, no single structural element appears to directly impose the observed Z-vs-A specificity, indicating that it might result from the combination of several features. Close investigation of the exonuclease domain suggests that the selectivity towards dZTP is not directly encoded in its active site.

**Figure 6.**
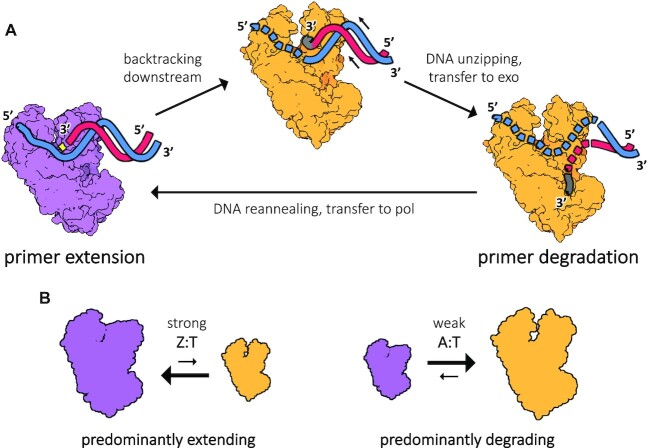
The exchange between the two functional modes of DpoZ, primer extension or degradation, could rely on a base pair-sensitive polymerase backtracking mechanism that leads to the proofreading step. (**A**) During DNA synthesis, the template strand (blue) and the primer strand (cherry red) are tightly held in place in the polymerase active site of DpoZ (purple, left). The primer is extended on the 3′-end with a free nucleotide (yellow) complementary to the upstream position on the template strand, in a stepwise cyclic process. Occasionally, incorporation errors or spontaneous uncoupling result in polymerase backtracking, when the extended primer's 3′-end (grey) retracts backward instead of being translocated in the forward direction (bright orange, middle) and the single-stranded part of the template strand becomes disordered (dotted lines; as in PDB 7B0G (62)). Eventually, the DNA primer strand's fraying end is sent to the exonuclease active site (bright orange, right). In this state, the 2–3 terminal nucleotides are subsequently removed from the primer. (**B**) In the proposed mechanism explaining DpoZ specificity, stronger Z:T bonds in the nascent DNA will permit the polymerase to stay predominantly in the extending mode. In contrast, weaker A:T bonds would lead to DNA uncoupling, polymerase backtracking and excision of the newly incorporated bases in the proofreading mode.

Consequently, we propose the following interpretation, coherent with all the results and the nature of A:T and Z:T base pairs: it is perhaps not the specific polymerase or exonuclease activity *per se* (i.e. their active site) which establishes the Z-vs-A preference, but rather the ability to promote the transition from the extending mode to the proofreading mode in the kinetic scheme of the polymerase reaction (Figure 6A). This ability has recently been shown to occur much more frequently than previously thought, including for correctly incorporated nucleotides (60). It relies in part on polymerase backtracking, which occurs just before the 3′-end of the newly synthetized strand is sent to the exonuclease active site; it is well described for DNA-dependent RNA polymerases (61), and structural evidence for this step has been found recently on DNA-dependent DNA polymerases (62). Such transitions – backtracking and DNA unzipping – would be naturally sensitive to the strength of the nascent base pair, differentiating between double and triple hydrogen bonds (Figure 6B). We propose that in ϕVC8 DpoZ the backtracking activity may be especially high, increasing the probability that a newly incorporated A base is sent to the exonuclease domain. In other words, the energy barrier needed to be crossed to reach the backtracking stage would be lowered in this PolA, whose function is to accept only triply hydrogen-bound base pairs, contrary to the typical members of the family.

However, pin-pointing exactly where in the structure this ability resides remains a difficult task. The feature that one should look for is subtle and dynamical in nature, probably involving an allosteric network of residues participating in the transition from the elongation (polymerase) mode of the enzyme to the proofreading (exonuclease) one. This communication pathway has been the subject of active research and is still unresolved and being investigated (63); it might actually be somewhat different in different polymerase families. A possible suggestion (that could be verified by protein engineering) concerns the ϕVC8 DpoZ insertion 117–127, which corresponds to the region 102–122 in T7 PolA, assumed to be implicated in DNA transfer between pol and exo sites (47). Additionally, this insertion is in contact with a loop containing the 356-TGR-358 motif, sometimes called pre-Motif A. We note that R358, a residue known to be important for the polymerase catalytic site (51), has a side-chain that changes rotamer between the thumb-exo open and closed forms (its movement can be noticed in the Supplementary Movie). To validate which residue(s) drive the transition from the extension to the proofreading modes, the structure of the ternary complex consisting of the polymerase with both the DNA duplex and the incoming dNTP is needed. So far, we could not obtain the crystals of the ternary complex with ϕVC8 DpoZ despite extensive screenings with exonuclease-deficient mutants.

The unique structural features observed in the crystallographic model of ϕVC8 DpoZ presented here are conserved in the sequences of other DpoZ polymerases from the ϕVC8-like clade. Thus, the functional characteristics that ensue can reasonably be extended to the whole group of close homologues, which includes acinetophage SH-Ab 15497. However, some of these features are absent in DpoZ from the Wayne-like clade: most prominently, these polymerases lack the insertion 2 on helix O and signature mutations L455 and G548 (5). Additionally, the mobile fragment including helices E1 and E2 seem to be shorter and does not follow the conservation pattern of the clade containing ϕVC8-like enzymes.

Concerning phage ϕJL001, its ATGC-DNA-processing PolA is indeed related to ϕVC8 DpoZ (26% sequence identity), but DpoZ of phage Wayne does not display any meaningful sequence homology with ϕJL001. Strikingly, phages with ϕVC8-like DpoZ as well as cyanophage S-2L (which lacks DpoZ) all possess a Z-cluster necessary for Z-to-A substitution composed of 3 genes (*datZ*, *mazZ*, *purZ*) (9) – in contrast, phages with Wayne-like DpoZ seem to replace *datZ* and *mazZ* with a single gene showing homology to dUTP phosphatases. It is therefore possible that a transfer of only the *purZ* gene, responsible for dZTP synthesis, have occurred between the two viral clades. In such a case, ϕVC8-like and Wayne-like DpoZ would have arisen separately from unrelated PolA proteins (one having a common ancestor with ϕJL001 PolA), and dZTP specificity would have been developed afterwards. As mentioned above, the Z-cluster is also absent in ϕJL001 genome (although we note there the presence of a thymidylate synthase-related gene), which could signify that its ancestor separated before the transfer of *purZ* to the ϕVC8-like clade, or that the *purZ* gene was subsequently lost.

An experimental structure of a Wayne-like DpoZ might be needed to resolve the question of DpoZ origin in the two phage clades. Such a structure could also be more prone to crystallize in binary and ternary complexes with double-stranded DNA and incoming dNTP. The resulting 3D models may then provide the key to fully elucidate the allosteric networks driving the specificity of DpoZ towards 2-aminoadenine, and thus enable engineering of cellular DNA polymerases towards enhanced assimilation and compartmentalization of ZTGC-DNA *in vivo*.

## DATA AVAILABILITY

The crystallographic data have been deposited to the PDB under the code 7PBK.

## Supplementary Material

gkab955_Supplemental_Files
