# Supplementary material for: Structural dynamics and determinants of 2-aminoadenine specificity in DNA polymerase DpoZ of vibriophage ϕVC8
Source: Nucleic Acids Res. 2021 Nov 9;49(20):11974–85. doi: 10.1093/nar/gkab955 (PMC8599892; doi:10.1093/nar/gkab955)
Supplement: gkab955_Supplemental_Files [file gkab955_supplemental_files.zip › article3_revised_suppl_data.pdf]

Structural dynamics and determinants of 2-aminoadenine specificity in DNA polymerase DpoZ of vibriophage ϕVC8

Dariusz Czernecki, Haidai Hu,
Filippo Romoli and Marc Delarue

Supplementary Information

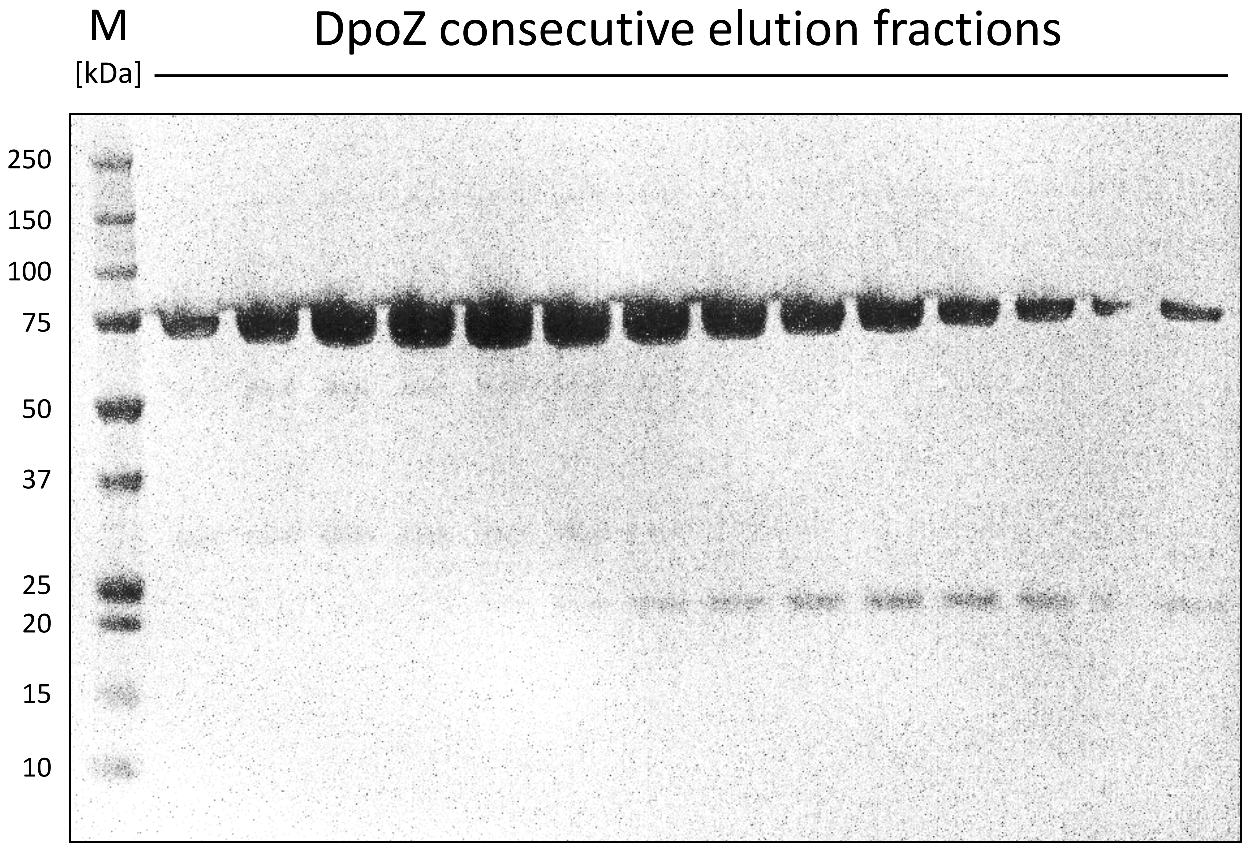

Supplementary Figure S1. SDS-PAGE gel of the DpoZ sample eluted in the final step of purification by gel filtration. Molecular weight markers (M) are on the first lane; consecutive fractions of DpoZ are in all the remaining lanes. The calculated weight of the 6-histidine-tagged DpoZ expressed in this assay is 72.7 kDa, which corresponds to the major band. Fractions on the right with low DpoZ content were discarded to avoid contaminants.

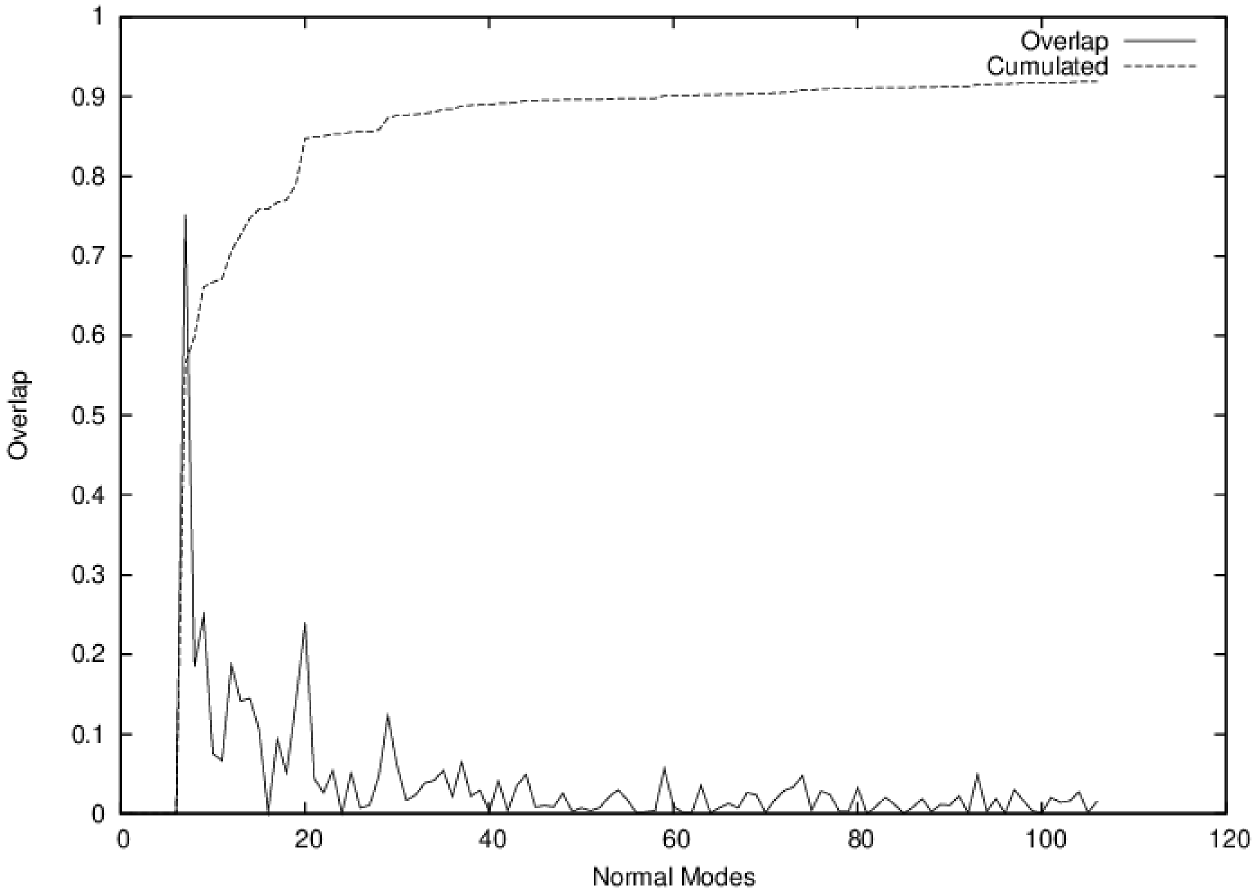

Supplementary Figure S2. Normal mode analysis of DpoZ movement between the thumb-exo open and closed states. In solid line are shown the overlap coefficients (normalized dot product) between i) each of the first 100 lowest-frequency normal mode vectors calculated with the coarse-grained Elastic Network Model (ENM) and ii) atomic displacement vectors between DpoZ thumb-exo open and closed forms. The cumulative overlap coefficient, counting from lowest-frequency modes, is showed with a dotted line. An overlap of 1 signifies complete explanation of the observed movement by normal modes. The first six modes represent rotation-translation modes with zero overlap, as expected. Only a handful (15-20) of lowest frequency normal modes derived from the model account for 80-90% of the transition between the two forms seen in the asymmetric unit.

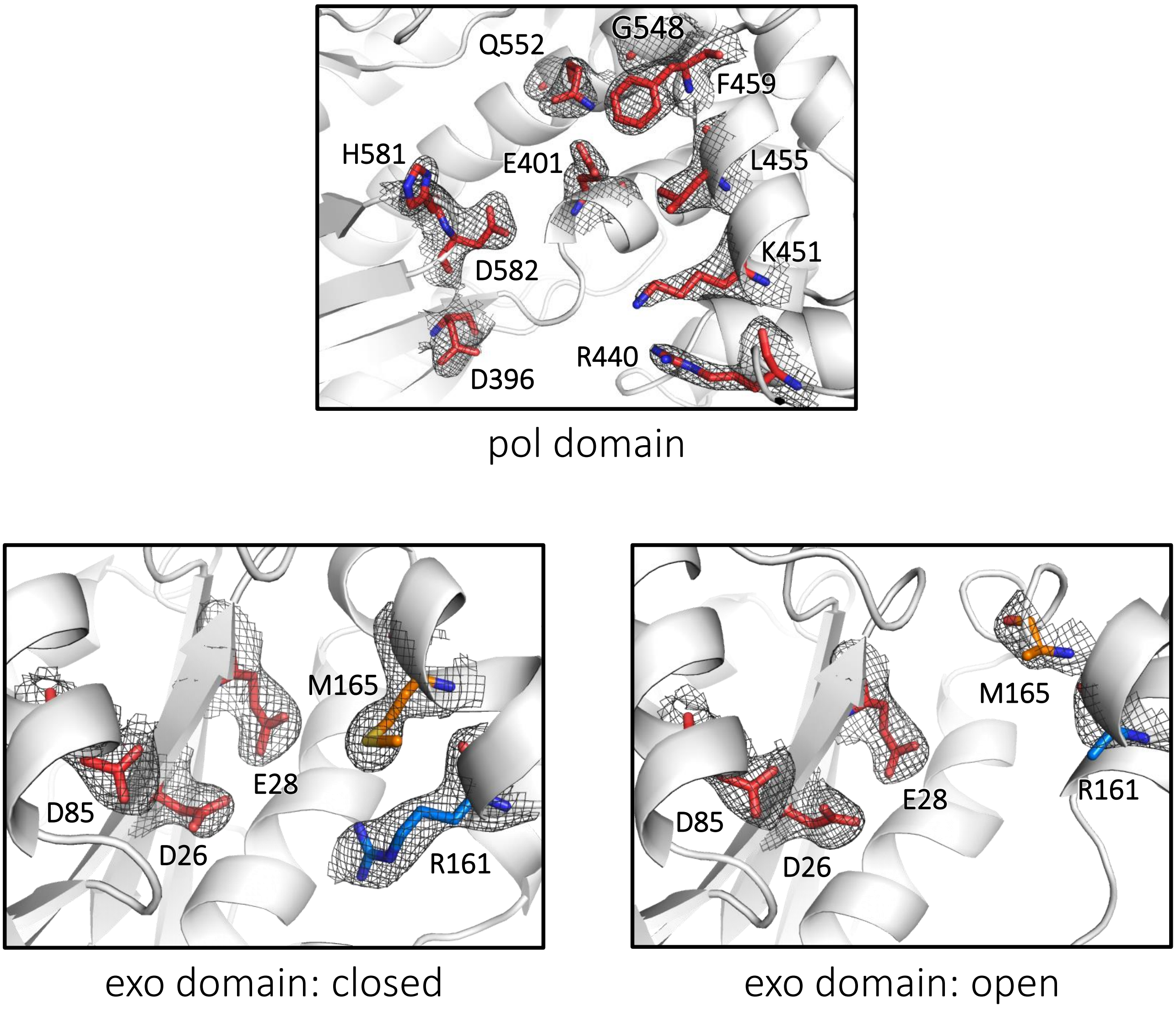

Supplementary Figure S3. Electron density of residues of interest around the polymerase and exonuclease active sites, in stick representation. The exonuclease catalytic pocket is shown in both open and closed states. Catalytic residues are coloured in red, R161 in blue and M165 in orange. The electron density outlining the residues is represented by a black mesh contoured at 1 sigma. In the open state, it does not extend over the sidechains of R161 and M165, leaving their side-chain atoms' position ambiguous.

***dpoZ* gene version**	**Nucleotide sequence**
wild-type	ATGAAGCTAGATTGGGAAAGAACAGGTCGACGGATGGGCTTCATCGACCTGTCTAAATACGAGGTTTGGTCTTATGACACAGAG<br>TGTACAGGACTCCAGTACAAAGTGGATAAAGTCTTTGGATTCAGCATCGCAACTCCCGACGGGCAGAGCGGATATTTCGACGTT<br>CGAGAGCAGCCTGAGTCGCTACAATGGCTTGCTGAACAGGTTGAACCCTACAAGGGAACAATCGTGTGTCATAACGCGTCATTT<br>GACTACAGAATGTCGCTACATTCCGGAATTAAGCTACCTCTTTCGCAGATTGATGATACGGGCATTAGAGCCTGCTGCATTAAT<br>GAGCATGAGTCAACTATTTTCCCTTGGACGCGTGGCAGAGCAGGTGACTATAGCCTTGACTACCTCGCAAAGAAATACGTGGGA<br>GCGCAGAAATACGCTGAGATTTATGATGAACTGGCTGCTCTCTTCGGAGGCAAAGCCACCCGCAAAACACAAATGCCGAATCTC<br>TATCGAGCGCCAAGTGGACTGGTAAGAAAGTACGCTTGTCCCGATGCTGAGCTTACTTTGGAATTGTGGCTTGAGCAGGAAGAA<br>CTCATAAAGAAACGTGGGCTTGAGAGAATAGTGGCATTCGAACGCAAGGTGATGCCGACTCTAATCAGAACCGAAGCTCGTGGG<br>GTGAGGGTTGACCTAGATTACGCTGAGCAAGCCATCTTCAAGATGGACGGCGTGGTGAGGGAGAACCAAGCCAAAATGTTTGCT<br>CTGGCAGGAAGGGAGTTTAACCCTAACTCGCCGAAGCAAGTTAGAGAGGTGTTCGGGGCGAAAGAGGAAGGCGGAGTCTGGAAA<br>TCGAGAGACGGCACGATTCTGGAGCGGACAGCGACTGGCAACCCGTGTTTGGATGCAGACGCACTGCGCTCAATGACAGACCCA<br>CTAGCCGCAGCAGTCTTGGAATTGAGAAGCAACATCAAGACCAAGGACACTTTCTTGGCCAAACACGTGGTGGAGCACAGCGTC<br>GGCGGTCGGGTTTACCCCAACATCAACCAGATGAAGGGGGAAGACGGCGGCACGGGGACAGGAAGATTGTCGTATACTGGACCC<br>GCATTGCAGCAGATTCCGTCCCGAAACAAAAGAATCGCAGCCATCATCAAGCCTGCGTTCTTGCCGGAAGAGGGTCAACTGTGG<br>TTGGATTCCGACATGGCGTCGTTTGAAGTCCGGATTTTCGCCCACTTGGTGGCGGCGTACAACCCTGCTATTGCAAAGGCGTAC<br>GCCGAGAACCCTGAACTCGACCTGCATCAGTGGGTGGGGGACTTGATGGGGATACCGCGCAACGCATCCTACTCAGGCCAACCT<br>AACGCCAAGCAAATGAACCTCGGCATGATTTTTAACCGTGGGGACGGCGCGGTGGCGGATAGCTTAGGCATGCCGTGGGAGTGG<br>TGCGAGTTCACCGACAAGAAAGGCGAACTAATTCGCTACAAAAAGGCTGGCCGAGAGGCGAAGTCAATCATCGCAGCTTACCAT<br>AGTCAAATCCAAGGGGTGAAGACTCTGGCAACGCGAGCACAAAAAATAGCCGAAGAAAGAGGCTGGATACAAACAGCCCACGGC<br>AGACGACTGCGGTTCCCCAACGGGTACAAGTCGTACAAAGCGTCAGGAATTTTGATTCAGGCAACCGCCGCTGACGAGAATAAA<br>GAAAACTGGCTGAGGATTGAAGACGCGTTGGGCAGCGACGGGTCGATGATTCTCAATACACACGATTCCTACTCAATGTCTGTC<br>GACGAGAATTGGAAACCAATATGGGAACGAGTCAAAAAAGCCGTTGAACGGCAGACTCTGCGTGTCCCTCTGTTATTGGAATTC<br>GATGGCGTCGGCAAGAATTGGGCCGAAGCAAAAGGATTAATCGATGTACATTAA
codon-optimized	ATGAAACTGGATTGGGAACGTACCGGTCGTCGTATGGGTTTTATTGATCTGAGCAAATATGAGGTTTGGAGCTATGCAACCGCA<br>TGTACCGGTCTGCAGTATAAAGTTGATAAAGTGTTTGGCTTCAGCATTGCAACACCGGATGGTCAGAGCGGTTATTTTGATGTT<br>CGTGAACAGCCGGAAAGCCTGCAGTGGCTGGCAGAACAGGTTGAACCGTATAAAGGCACCATTGTTTGTCATAATGCAAGCTTT<br>GCATATCGCATGAGCCTGCATAGCGGTATTAAACTGCCGCTGAGCCAGATTGATGATACCGGCATTCGTGCATGTTGTATCAAT<br>GAACATGAAAGCACCATTTTTCCGTGGACACGTGGTCGTGCCGGTGATTATAGCCTGGATTATCTGGCCAAAAAATACGTTGGT<br>GCACAGAAATATGCCGAGATCTATGATGAACTGGCAGCACTGTTTGGTGGTAAAGCAACCCGTAAAACCCAGATGCCGAATCTG<br>TATCGTGCACCGAGCGGTCTGGTTCGTAAATATGCATGTCCGGATGCAGAACTGACCCTGGAACTGTGGCTGGAACAAGAAGAA<br>CTGATCAAAAAACGTGGTCTGGAACGTATTGTTGCCTTTGAACGTAAAGTTATGCCGACACTGATTCGTACCGAAGCACGTGGT<br>GTTCGTGTTGATCTGGATTATGCCGAACAGGCAATCTTTAAAATGGATGGTGTTGTGCGTGAAAACCAGGCAAAAATGTTTGCA<br>CTGGCAGGTCGTGAATTTAATCCGAATAGCCCGAAACAGGTTCGTGAAGTTTTTGGTGCAAAAGAAGAAGGCGGAGTTTGGAAA<br>AGCCGTGATGGCACCATTCTGGAACGCACCGCAACCGGTAATCCGTGTCTGGATGCCGATGCACTGCGTAGCATGACCGATCCG<br>CTGGCAGCAGCAGTTCTGGAACTGCGTAGTAACATTAAAACCAAAGATACCTTTCTGGCCAAGCATGTTGTTGAACATAGCGTT<br>GGTGGTCGTGTTTATCCGAACATTAATCAGATGAAAGGTGAAGATGGTGGCACCGGTACAGGTCGTCTGAGCTATACCGGTCCG<br>GCACTGCAGCAGATTCCGAGCCGTAATAAACGTATTGCAGCAATTATCAAACCGGCATTTCTGCCGGAAGAGGGTCAGCTGTGG<br>TTAGATAGCGATATGGCCAGCTTTGAAGTTCGTATTTTTGCACATCTGGTGGCAGCATATAATCCGGCAATTGCAAAAGCATAT<br>GCAGAAAATCCGGAACTGGATCTGCATCAGTGGGTTGGTGATCTGATGGGTATTCCGCGTAATGCCAGCTATAGCGGTCAGCCG<br>AATGCAAAACAAATGAATCTGGGCATGATTTTTAACCGTGGTGATGGTGCAGTTGCAGATAGCCTGGGTATGCCGTGGGAATGG<br>TGTGAATTTACCGATAAAAAAGGTGAACTGATCCGCTACAAAAAAGCCGGTCGTGAAGCAAAAAGCATTATTGCAGCCTATCAT<br>TCACAGATTCAGGGTGTGAAAACCCTGGCAACCCGTGCGCAGAAAATTGCGGAAGAACGTGGTTGGATTCAGACCGCACATGGT<br>CGTCGTCTGCGCTTTCCGAATGGTTATAAAAGCTATAAAGCAAGCGGCATTCTGATTCAGGCAACCGCAGCAGATGAAAATAAA<br>GAAAATTGGCTGCGTATTGAAGATGCCCTGGGTAGTGATGGTAGCATGATTCTGAATACACATGATAGCTATAGCATGAGCGTG<br>GATGAAAATTGGAAACCGATTTGGGAACGCGTTAAAAAAGCAGTTGAACGTCAGACCCTGCGTGTTCCGCTGCTGCTGGAATTT<br>GATGGTGTGGGTAAAAATTGGGCAGAAGCAAAAGGTCTGATCGATGTGCATTAA

Supplementary Table S1. Nucleotide sequences of ϕVC8 wild-type and codon-optimized *dpoZ* genes.

Oligonucleotide name	Sequence (5’-3’)
T_{24} overhang template	TTTTTTTTTTTTTTTTTTTTTTTTAACAAGGCTAATGCG
Primer	CGCATTAGCCTTGTT

Supplementary Table S2. Oligonucleotides used for the polymerase assay.

**Protein structure**	**ϕVC8 DpoZ**
PDB ID	7PBK
Cell parameters	
Space group	$P\ 2_1\ 2_1\ 2_1$
a, *b*, *c* (Å)	120.16, 158.44, 79.95
α, *β*, *γ* (°)	90.0, 90.0, 90.0
Solvent content (%)	53.0
Data statistics	
Resolution (Å)	48.35 - 2.79 (2.87 - 2.79)
Wavelength (Å)	1.6926
Rmerge (%)	35.2 (261.7)
Completeness (%)	99.4 (92.7)
Multiplicity	26.5 (23.5)
$I/\sigma(I)$	9.7 (1.4)
$CC_{1/2}$	0.996 (0.927)
Refinement	
Resolution (Å)	48.35 - 2.80
Unique reflections	38,233
R_{work}/R_{free} (%)	19.02/25.65
No. of non-hydrogen atoms	
Protein	9575
Water	133
Protein geometry	
RMSD - bond lengths (Å)	0.008
RMSD - bond angles (°)	0.95

**Protein structure**	**ϕVC8 DpoZ**
PDB ID	7PBK
Protein geometry (cont.)	
Ramachandran favored/outliers (%)	95.28/0.00
Rotamers favored/poor (%)	90.27/0.00
Clashscore	7.75
B-factors (Å^2)	
TLS	1 group
Protein	56.39
Water	51.56

Supplementary Table S3. Diffraction data and Model Refinement statistics. Numbers in parenthesis refer to the highest-resolution shell.

Supplementary movie caption

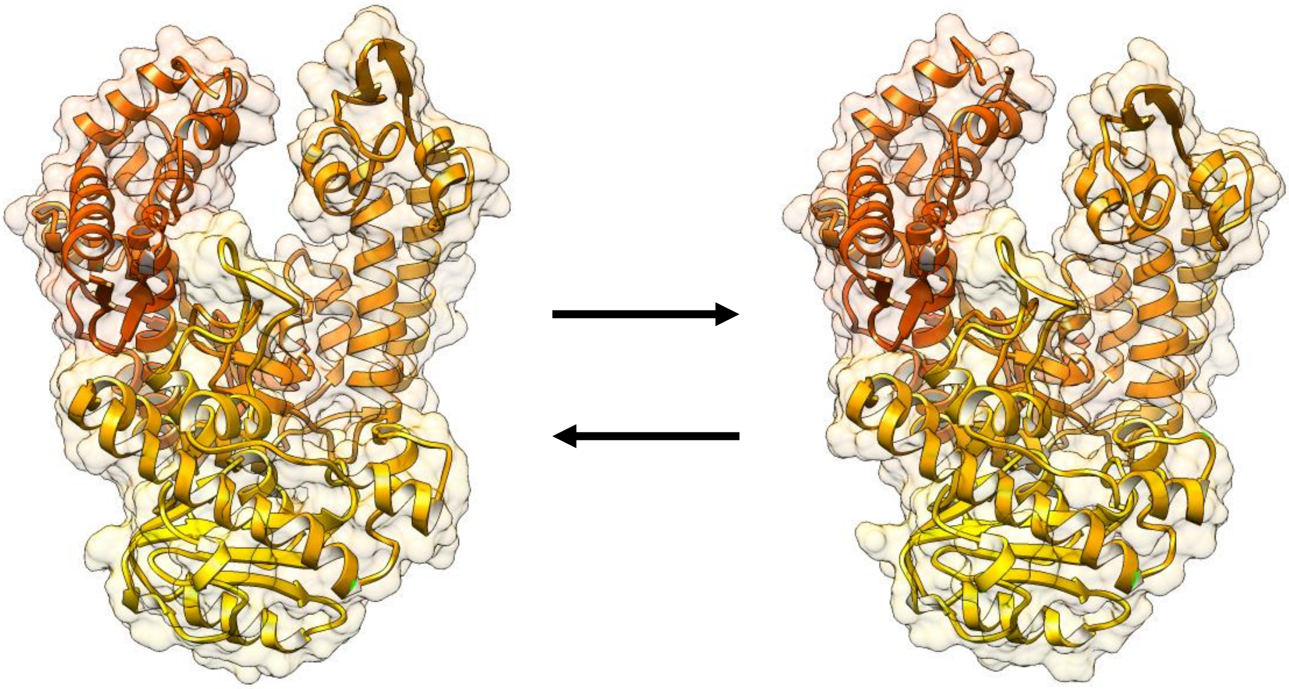

Supplementary Movie S1. Transition between ϕVC8 DpoZ thumb-exo open and closed states. The polymerase is displayed in ribbon-surface representation and coloured with a yellow-dark orange gradient. The interpolation of DpoZ movement was made in Chimera, using the corkscrew method.
